# Impact of PBP3 insertions and β-lactamase-encoding genes on the susceptibility of *Escherichia coli* isolates belonging to high-risk clones against aztreonam-avibactam and comparator agents

**DOI:** 10.1128/aac.00129-26

**Published:** 2026-09-09

**Authors:** Mariana Castanheira, John H. Kimbrough, Maura Karr, Helio S. Sader

**Affiliations:** 1 Element Materials Technology (JMI Laboratories), North Liberty, Iowa, USA; University of Fribourg, Fribourg, Switzerland

**Keywords:** PBP3 insertions, β-lactamases, β-lactam resistance, β-lactam/β-lactamase inhibitor combinations

## Abstract

We evaluated the presence and phenotypic impact of PBP3 insertions YRIN (*n* = 44) and YRIK (*n* = 18) among 124 *Escherichia coli* isolates belonging to high-risk lineages selected from 1,471 isolates displaying resistance to β-lactams. PBP3 insertions were detected among 62 isolates. YRIN insertions were detected among isolates belonging to ST167 (*n* = 23), ST361 (*n* = 7), ST410 (*n* = 10), ST617 (*n* = 1), and ST648 (*n* = 3), while YRIK insertions were observed in ST405 (*n* = 9), ST410 (*n* = 5), and ST617 (*n* = 4). Aztreonam-avibactam inhibited 90.3% of all isolates from high-risk clones and 80.6% of the isolates harboring PBP3 insertions when applying the EUCAST breakpoints. Aztreonam-avibactam inhibited 66.7% of the YRIK-harboring isolates (MIC_50/90_, 2/16 mg/L) and 86.4% of the ones harboring YRIN insertions (MIC_50/90_, 1/8 mg/L); however, the susceptibilities to other β-lactams were lower among YRIN isolates (25.6%–60.5%) compared to YRIK (55.6%–88.9%). Overall, ST617 was more resistant to all agents when compared to other STs, regardless of PBP3 insertions or the presence of other examined resistance mechanisms. Acquired β-lactamase genes and OmpC deleterious alterations were present in all isolates. CMY-42, CMY-141, and CMY-145 that have been associated with elevated aztreonam-avibactam MICs were detected among 13 isolates, and 4 (30.8%) were susceptible to this combination. All isolates harboring *bla*_NDM-5_ (*n* = 37) possessed PBP3 insertions. Aztreonam-avibactam inhibited 83.8% of these isolates. Resistance to aztreonam-avibactam is multifactorial, and singular resistance mechanisms are unable to elevate MIC values to resistant categories. Additionally, lineage-specific contributions to resistance play an important role in the overall development of β-lactam resistance, although these mechanisms appear to function outside the known repertoire of resistance-conferring mutations and warrant further study.

## INTRODUCTION

Aztreonam is a monobactam that has been used in clinical practice since the late 1980s (1). The primary target for aztreonam is penicillin-binding protein (PBP) 3, and this β-lactam exhibits poor affinity for other PBPs (2). Alterations in PBP3 have been demonstrated to cause resistance to aztreonam; however, the primary cause of resistance to aztreonam and other β-lactams in clinical isolates is the production of β-lactamases (3). To overcome the presence of β-lactamases, aztreonam was combined with the diazabicyclooctane inhibitor avibactam. Different from other newer β-lactam/β-lactamase inhibitor (BL/BLI) combinations, aztreonam-avibactam has activity against isolates producing metallo-β-lactamases (MBLs) (4, 5), as aztreonam is not hydrolyzed by these enzymes, and avibactam protects aztreonam from the degradation of other β-lactamases present in the cell (5). Despite this advantage, avibactam cannot protect aztreonam from non-β-lactamase-mediated resistance mechanisms, such as alterations affecting cell permeability, extrusion of the agent from the cell by efflux, or target modifications.

Studies have reported that elevated aztreonam-avibactam MIC results in *Escherichia coli* isolates were associated with the insertions YRIN and YRIK in position 333 of PBP3 (6–11). The impact of these PBP3 insertions is not limited to aztreonam-avibactam; they have also been associated with reduced susceptibility to other β-lactams that target PBP3, including the cephalosporins and the siderophore-cephalosporin cefiderocol (7). However, a large body of evidence has been generated indicating that a combination of multiple resistance mechanisms is required to increase the aztreonam-avibactam MIC values to a resistant category and that resistant isolates are uncommon (6, 12–17). This is corroborated by large-scale surveillance studies demonstrating the activity of aztreonam-avibactam to be >98.4% among global collections of Enterobacterales isolates (99.8%–99.9% susceptibility) (12–14), carbapenem-resistant Enterobacterales (CREs, 98.4%–99.6%) (16, 17), and MBL-producing isolates (100.0%) (15). In a recent study evaluating 490 MBL-producing Enterobacterales, among 40 *E. coli* isolates producing MBLs, 30 carried PBP3 insertions YRIN or YRIK (15). All these isolates were susceptible to aztreonam-avibactam when applying the European Committee on Antimicrobial Susceptibility Testing (EUCAST) and US Food and Drug Administration (FDA) susceptibility breakpoints.

A study by Long et al. examined >159,000 *E*. *coli* genomes in the National Center for Biotechnology Information database and found that PBP3 inserts are common among certain sequence types (STs) (18). The insertions were detected among isolates from numerous countries and were more commonly associated with certain STs, namely, ST410, ST167, and ST405. These STs are considered high-risk clones, and >80% harbored MBL genes encoding NDM variants (18). Unfortunately, data on the susceptibility of aztreonam-avibactam against those isolates were not available.

In this study, we evaluated 1,471 *E. coli* isolates collected from 2022 to 2024, and their susceptibility was tested as part of the aztreonam-avibactam surveillance program. Isolates displaying elevated MIC values for aztreonam, cephalosporins, and/or carbapenems were submitted to whole genome sequencing. Among these, isolates belonging to high-risk clonal STs 167, 361, 405, 410, 617, and 648 were examined for the presence of PBP3 alterations and other β-lactam resistance mechanisms. The susceptibility of these isolates against aztreonam-avibactam and other β-lactam and BL/BLI combinations was analyzed to assess the impact of resistance mechanisms on antimicrobial activity.

## MATERIALS AND METHODS

### Bacterial isolates

A total of 11,617 *E. coli* isolates were collected from 2022 to 2024 in hospitals located in Asia-Pacific (17 hospitals), Europe (40), and Latin America (10). These isolates were collected as part of a global surveillance study monitoring prospectively consecutive non-duplicated isolates collected from bloodstream, urinary tract, intra-abdominal, skin, and skin structure infections and patients hospitalized with pneumonia. Bacterial isolates determined to be the cause of infection by local clinical and/or microbiological criteria and only one isolate per patient episode were included in this investigation. Species identification was confirmed by MALDI-TOF MS using the Bruker Daltonics MALDI Biotyper (Billerica, MA) following the manufacturer’s instructions.

### Antimicrobial susceptibility testing

Antimicrobial susceptibility testing was performed by reference broth microdilution methods according to Clinical and Laboratory Standards Institute (CLSI) procedures (19). Cefiderocol was tested in iron-depleted cation-adjusted Mueller-Hinton broth. Avibactam was tested at a fixed 4 mg/L concentration when combined with aztreonam or ceftazidime, and taniborbactam was tested at a fixed 4 mg/L when combined with cefepime. Zidebactam was tested in a 1:1 ratio with cefepime. Antimicrobial agents were procured from Pfizer (avibactam; New York, NY, USA), MedChem Express (cefiderocol, taniborbactam, and zidebactam; Monmouth Junction, NJ, USA), USP Pharmacopeia (Rockville, MD, USA), or Sigma-Aldrich (St. Louis, MO, USA).

Quality control (QC) testing was performed to ensure proper test conditions by concomitantly testing the following QC strains: *Escherichia coli* ATCC 25922 and NCTC 13353, *Klebsiella pneumoniae* ATCC 700603 and ATCC BAA-1705, and *Pseudomonas aeruginosa* ATCC 27853. EUCAST and US FDA clinical breakpoints were used for the interpretation of susceptibility rates (20). The susceptible breakpoint for cefepime tested alone was applied to cefepime-taniborbactam and cefepime-zidebactam for comparison purposes.

### Characterization of β-lactam resistance mechanisms

Among the isolates collected, a total of 1,471 *E. coli* isolates displaying MIC values of >2 mg/L for two of the following agents—aztreonam, ceftazidime, cefepime, and/or non-susceptible MIC values from imipenem and/or meropenem according to the CLSI breakpoint criteria (21)—were subjected to whole genome sequencing. Total genomic DNA was used as input material for library construction, and sequencing libraries were prepared using Illumina DNA Prep library construction protocol and index kit (Illumina, San Diego, CA, USA) following the manufacturer’s instructions. Sequencing was performed using an Illumina NextSeq 1000/2000 sequencer. FASTQ format files for each sample set were assembled independently as previously described. Multilocus sequence typing was performed *in silico* (22). Isolates belonging to STs 167, 361, 405, 410, 617, and 648 were submitted to analysis to detect the presence of acquired β-lactamases and alterations in PBPs, outer membrane proteins (OMPs), and the efflux regulator AcrR. Deleterious mutations in OmpC, OmpF, and AcrR are defined here as those alterations resulting in large, disruptive effects on protein structure, including insertions, deletions, nonsense mutations, frameshift mutations, or the lack of an identifiable coding sequence for those proteins. Potential matches for β-lactamase and selected genes were generated with the criteria of >94% identity and 40% minimum coverage length when compared to reference sequences. PBP3, OmpC, OmpF, and AcrR sequences were compared to those of *E. coli* ATCC 25922.

## RESULTS

### Sequence types and β-lactam resistance mechanisms

Among a collection of 1,471 *E. coli* isolates that were submitted to whole genome sequencing due to resistance to aztreonam, ceftazidime, ceftriaxone, cefepime, and/or carbapenems, 124 (8.4%) isolates belonged to STs 167 (*n* = 33; includes one single locus variant [SLV], defined as an ST differing from the listed ST at a single MLST locus), 361 (*n* = 10), 405 (*n* = 18, includes 1 SLV), 410 (*n* = 20), 617 (*n* = 16), and 648 (*n* = 27, includes 1 SLV).

**Fig 1:**
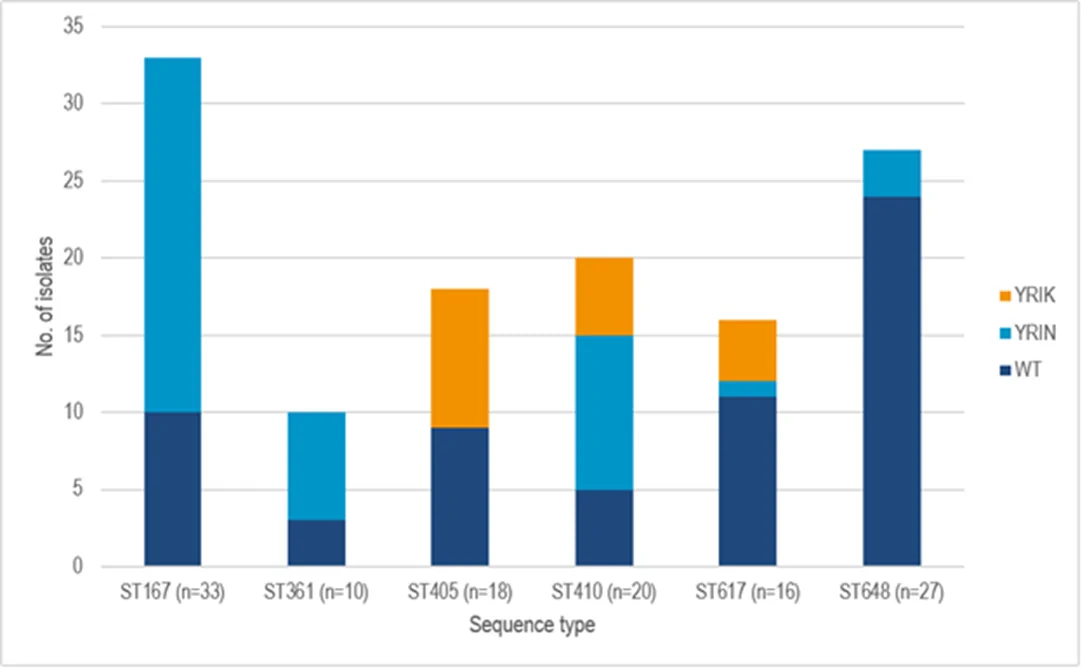
Distribution of PBP3 inserts among *E. coli* isolates from STs 167, 361, 405, 410, 617, and 648.

Further genetic analysis revealed that PBP3 insertions YRIK or YRIN following position 333 were present in 62 (50.0%) isolates—18 and 44 isolates harbored YRIK or YRIN, respectively. PBP3 YRIN insertions were detected among 23/33 isolates belonging to ST167, 7/10 of ST361, 10/20 of ST410, 1/16 of ST617, and 3/27 of ST648 (Fig. 1). Isolates harboring PBP3 YRIK insertions belonged to only three STs: 9/18 of the ST405 isolates, 5/20 of ST410, and 4/16 of ST617. Discrete amino acid changes in the PBP3 sequences were noted among 124 isolates, namely, T75A (1 isolate), E149D (99 isolates), T233A (124 isolates), Q227H (56 isolates), V332I (91 isolates), A317T/V (2 isolates), E349K (44 isolates), I532L (44 isolates), L369F (1 isolate), A413V (20 isolates), and S473I (1 isolate; Table S1). Interestingly, regardless of the ST, alterations E149D, T233A, V332I, E349K, and I532L were noted in all isolates harboring YRIN PBP3 insertions, and E149D, T233A, V332I, and A413V were noted in all isolates harboring YRIK PBP3 insertions.

Isolates harboring PBP3 insertions were recovered from 11 countries with multiple STs represented in most countries: Australia (2 isolates from ST167, 1 from ST410, and 1 from ST617), France (1 from ST167 and 1 from ST361), Germany (1 from ST167 and 2 from ST648), Israel (1 from ST361 and 1 from ST410), Italy (1 from ST405 and 1 from ST410), Mexico (17 from ST167, 4 from ST361, 8 from ST405, and 2 from ST410), Panama (2 from ST410 and 1 from ST617), Philippines (2 from ST167, 1 from ST361, and 1 from ST410), Taiwan (1 from ST410 and 3 from ST617), Thailand (2 from ST410), and Turkey (4 from ST410 and 1 from ST648; Table 1).

**TABLE 1:** Distribution of *E. coli* isolates from STs 167, 361, 405, 410, 617, and 648 by country^*a*^

Country	No. of overall isolates (no. of isolates with PBP3 insertions)	No. of isolates by ST (no. of isolates with PBP3 YRIN/YRIK insertions)					
		ST167	ST361	ST405	ST410	ST617	ST648
All	124 (62)	33 (23/0)	10 (7/0)	18 (0/9)	20 (10/5)	16 (1/4)	27 (3/0)
Australia	5 (4)	2 (2/0)			1 (1/0)	1 (0/1)	1
Belgium	6 (0)	2					4
Brazil	3 (0)		1		1	1	
Chile	1 (0)						1
Czech Republic	1 (0)			1			
France	9 (2)	2 (1/0)	1 (1/0)	2		1	3
Germany	12 (3)	2 (1/0)		3	2		5 (2/0)
Greece	1 (0)			1			
Israel	6 (2)		1 (1/0)	1	1 (1/0)	3	
Italy	4 (2)			1 (0/1)	2 (1/0)		1
Korea	3 (0)					2	1
Mexico	35 (31)	19 (17/0)	5 (4/0)	8 (0/8)	2 (0/2)	1	
Panama	5 (3)				3 (2/0)	1 (1/0)	1
Philippines	5 (4)	2 (2/0)	1 (1/0)		1 (1/0)		1
Spain	2 (0)					1	1
Sweden	2 (0)						2
Switzerland	2 (0)						2
Taiwan	4 (4)				1 (1/0)	3 (0/3)	
Thailand	2 (2)				2 (2/0)		
Turkey	15 (5)	4	1	1	4 (1/3)	2	3 (1/0)
UK	1 (0)						1

^*a*^ Isolates harboring PBP3 insertions YRIN and YRIK are displayed in parentheses.

**Fig 2:**
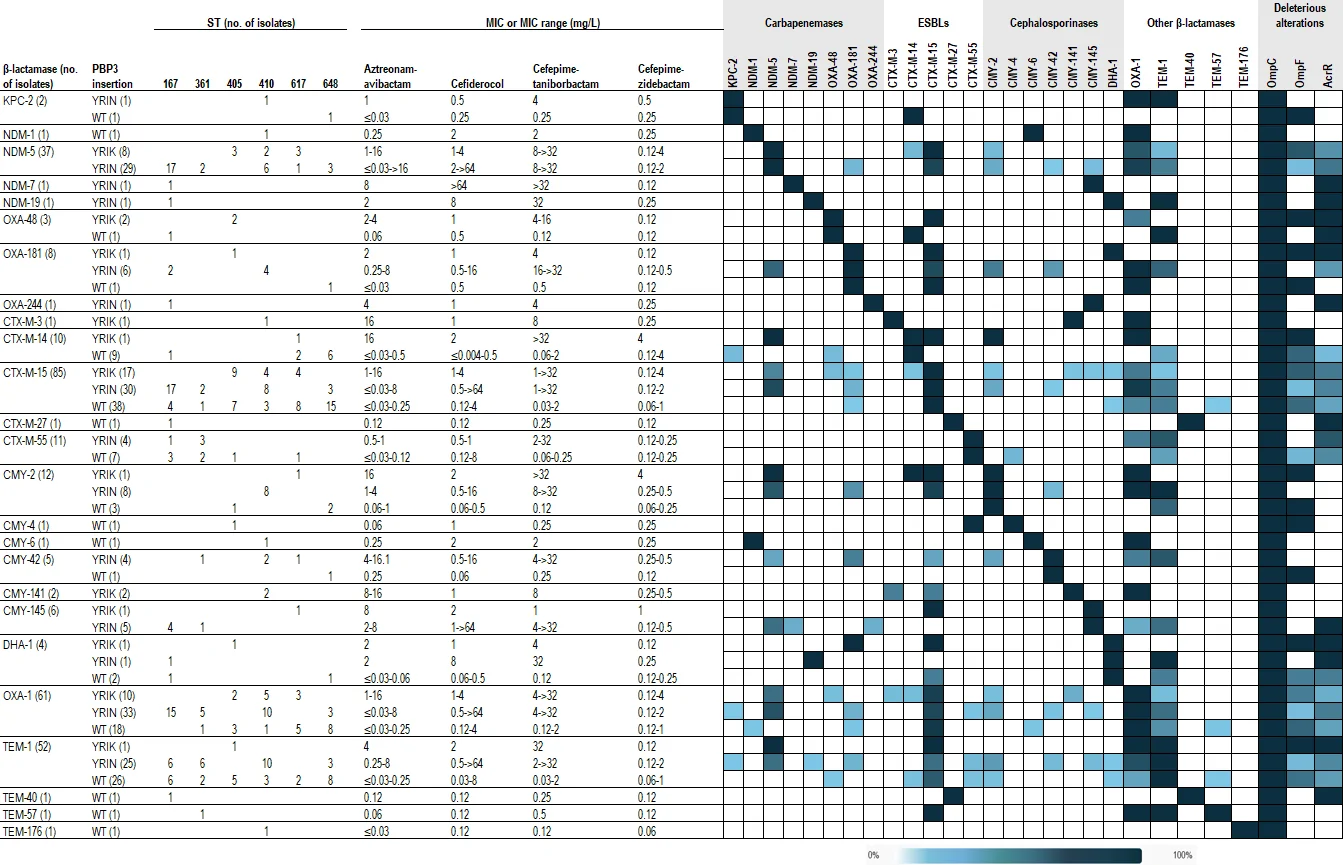
Activity of aztreonam-avibactam and key comparator agents against *E. coli* isolates belonging to high-risk clones producing β-lactamases with and without PBP3 inserts. The heatmap displays the percentage of other β-lactam resistance mechanisms.

Acquired β-lactamase genes were detected in all 124 isolates and included genes encoding 25 enzymes/variants (Fig. 2) in 49 enzyme combinations (Table S2). Carbapenemase-encoding genes were observed among 50 isolates, and 46 of the isolates carrying those genes also harbored PBP3 insertions. Notably, *bla*_NDM-5_ was the most common carbapenemase-encoding gene (*n* = 37) and was only detected among isolates carrying PBP3 insertions (29 isolates harboring YRIN and 8 harboring YRIK). Other carbapenemase-encoding genes included *bla*_OXA-181_ (*n* = 8; 1 YRIK, 6 YRIN, and 1 displaying a wild-type [WT] PBP3 sequence at position P333_Y334), *bla*_OXA-48_ (*n* = 3; 2 YRIK and 1 WT), *bla*_KPC-2_ (*n* = 2; 1 YRIN and 1 WT), one isolate carrying *bla*_NDM-1_ that was WT for PBP3, and one isolate carrying each of *bla*_NDM-7_, *bla*_NDM-19_, and *bla*_OXA-244_, all harboring a PBP3 YRIN insertion.

A total of 107 isolates carried ESBL-encoding genes, all *bla*_CTX-M_. The gene encoding CTX-M-15 was noted in 85 isolates, including isolates carrying PBP3 YRIN (*n* = 30) and YRIK (*n* = 17) insertions and isolates with WT (*n* = 38) sequences. Moreover, *bla*_CTX-M-14_, *bla*_CTX-M-55_, *bla*_CTX-M-3_, and *bla*_CTX-M-27_ were detected in 10, 11, 1, and 1 isolates, respectively. PBP3 insertions were noted among only one isolate harboring *bla*_CTX-M-14_ (YRIK) that also carried *bla*_CTX-M-15_, four isolates carrying *bla*_CTX-M-55_ (YRIN), and the isolates harboring *bla*_CTX-M-3_ (YRIK).

CMY cephalosporinases were identified among 26 isolates, with 1 isolate carrying two distinct *bla*_CMY_ alleles (*bla*_CMY-2_ and *bla*_CMY-42_). These included *bla*_CMY-2_ (*n* = 12; 1 YRIK, 8 YRIN, and 3 WT), *bla*_CMY-4_ (*n* = 1, WT), *bla*_CMY-6_ (*n* = 1, WT), and variants that have been associated with elevated aztreonam-avibactam MIC values (9, 10, 23, 24): *bla*_CMY-42_ (*n* = 5; 4 YRIN and 1 WT), *bla*_CMY-141_ (*n* = 2, 2 YRIK), and *bla*_CMY-145_ (*n* = 6; 1 YRIK and 5 YRIN). Other β-lactamases observed among the isolates belonging to high-risk clones were *bla*_DHA-1_ (*n* = 4; 1 YRIK, 1 YRIN, and 2 WT), *bla*_OXA-1_ (*n* = 61; 10 YRIK, 33 YRIN, and 18 WT), *bla*_TEM-1_ (*n* = 52; 1 YRIK, 25 YRIN, and 26 WT), *bla*_TEM-40_, *bla*_TEM-57_, and *bla*_TEM-176_, all 1 PBP3 WT isolate.

All 124 isolates harbored deleterious alterations, including insertions/deletions, nonsense mutations, or absence of the gene encoding OmpC. OmpF deleterious alterations were observed among 50 isolates: 20/43 isolates harboring YRIN PBP3 insertions, 7/18 harboring YRIK, and 22/63 not displaying these insertions. A similar number of isolates (*n* = 48) possessed deleterious mutations in the efflux pump regulator AcrR, with over 50% of these isolates belonging to ST167 (*n* = 28). These resistance mechanisms were detected in complex combinations (Fig. 2). Alterations in OmpF and AcrR were distributed across isolates in the full range of aztreonam-avibactam MIC values.

### Susceptibility profiles

The susceptibility of all β-lactams and BL/BLIs was reduced for the 62 isolates harboring PBP3 inserts when compared to all 124 *E. coli* isolates analyzed (Table 2). Aztreonam-avibactam was active against 90.3% of the high-risk clone isolates (MIC_50/90_, 0.25/4 mg/L) and 80.6% of the isolates harboring PBP3 insertions (MIC_50/90_, 2/8 mg/L). Comparably, isolates from these high-risk clones displaying PBP3 wild-type sequences displayed high aztreonam-avibactam susceptibility (100% susceptible; MIC_50/90_, 0.06/0.25 mg/L; data not shown). All other clinically available agents displayed lower susceptibility rates against all isolates from high-risk clones and, similarly to aztreonam-avibactam, a decrease in activity against isolates harboring PBP3 insertions. Cefiderocol inhibited 83.1% vs 69.4%, cefepime-taniborbactam 49.2% vs 4.8%, ceftazidime-avibactam and meropenem-vaborbactam 70.2% vs 41.9%, imipenem-relebactam 67.7% vs 37.1%, meropenem 66.1% vs 35.5%, and imipenem 66.9% vs 35.5% of all high-risk clone isolates vs isolates harboring PBP3 insertions, respectively. Piperacillin-tazobactam, ceftolozane-tazobactam, aztreonam, ceftriaxone, ceftazidime, and cefepime displayed no activity against isolates carrying PBP3 insertions (0% susceptible, data not shown). All isolates included in this analysis, including the ones carrying PBP3 insertions, displayed MIC values at ≤4 mg/L for cefepime-zidebactam and were considered susceptible when applying the cefepime alone breakpoint for comparison purposes. Despite the activity of this BL/BLI, isolates harboring PBP3 insertions displayed higher MIC values against this combination (Fig. 2).

**TABLE 2:** Activity of aztreonam-avibactam and key comparator agents against *E. coli* isolates belonging to high-risk clones displaying PBP3 insertions and stratified by sequence type^*a*^

	Aztreonam-avibactam				Cefiderocol				Cefepime-taniborbactam				Ceftazidime-avibactam			
Isolates (no. tested)	MIC_50_	MIC_90_	MIC range	%S^*b*^	MIC_50_	MIC_90_	MIC range	%S^*b*^	MIC_50_	MIC_90_	MIC range	%S^*b*^	MIC_50_	MIC_90_	MIC range	%S^*b*^
All isolates (*n* = 124)	0.25	4	≤0.03 to >16	90.3	1	4	≤0.004 to >64	83.1	2	>32	0.03 to >32	49.2	0.5	>32	≤0.015 to >32	70.2
PBP3 insertions (*n* = 62)	2	8	≤0.03 to >16	80.6	2	8	0.5 to >64	69.4	16	>32	1 to >32	4.8	>32	>32	≤0.015 to >32	41.9
YRIN (*n* = 44)	1	8	≤0.03 to >16	86.4	2	16	0.5 to >64	61.4	16	>32	1 to >32	4.5	>32	>32	≤0.015 to >32	36.4
YRIK (*n* = 18)	2	16	1 to 16	66.7	2	4	1 to 4	88.9	8	>32	1 to >32	5.6	4	>32	0.5 to >32	55.6
167 (*n* = 33)	0.25	2	≤0.03 to 8	93.9	2	>64	0.06 to >64	69.7	16	>32	0.06 to >32	36.4	2	>32	≤0.015 to >32	51.5
167 PBP3 (*n* = 23)	0.5	4	≤0.03 to 8	91.3	2	>64	1 to >64	56.5	16	>32	1 to >32	8.7	>32	>32	≤0.015 to >32	30.4
361 (*n* = 10)	1	8	0.06 to 8	80	0.5	2	0.12 to 8	90	4	32	0.06 to >32	30.0	1	>32	0.25 to >32	80
361 PBP3 (*n* = 7)	1		0.5 to 8	71.4	0.5		0.5 to 2	100	16		2 to >32	0.0	2		0.5 to >32	71.4
405 (*n* = 18)	0.12	4	≤0.03 to 4	100	1	2	0.25 to 2	100	2	32	0.03 to 32	44.4	0.25	>32	≤0.015 to >32	83.3
405 PBP3 (*n* = 9)	2		1 to 4	100	2		1 to 2	100	16		4 to 32	0.0	2		0.5 to >32	66.7
410 (*n* = 20)	1	8	≤0.03 to 16	85	1	8	0.12 to 16	75	8	32	0.06 to >32	20.0	4	>32	≤0.015 to >32	55
410 PBP3 (*n* = 15)	2	8	1 to 16	80	2	8	0.5 to 16	66.7	16	>32	4 to >32	0.0	>32	>32	1 to >32	46.7
617 (*n* = 16)	0.06	16	≤0.03 to >16	68.8	1	4	0.06 to 16	81.2	0.25	>32	0.06 to >32	75.0	0.12	>32	≤0.015 to >32	75
617 PBP3 (*n* = 5)	8		8 to >16	0	4		2 to 16	40	>32		1 to >32	20.0	>32		4 to >32	20
648 (*n* = 27)	0.06	0.5	≤0.03 to 4	100	0.5	2	≤0.004 to 8	92.6	0.25	16	0.06 to >32	81.5	0.25	>32	≤0.015 to >32	88.9
648 PBP3 (*n* = 3)	0.5		0.5 to 4	100	2		2 to 8	66.7	>32		16 to >32	0.0	>32		>32 to >32	0

	Meropenem-vaborbactam				Imipenem-relebactam				Meropenem				Imipenem			
Isolates (no. tested)	MIC_50_	MIC_90_	MIC range	%S^*b*^	MIC_50_	MIC_90_	MIC range	%S^*b*^	MIC_50_	MIC_90_	MIC range	%S^*b*^	MIC_50_	MIC_90_	MIC range	%S^*b*^
All isolates (*n* = 124)	0.03	>32	≤0.015 to >32	70.2	0.12	16	0.06 to >16	67.7	0.06	>32	≤0.015 to >32	66.1	0.25	>8	≤0.12 to >8	66.9
PBP3 insertions (*n* = 62)	32	>32	≤0.015 to >32	41.9	4	16	0.06 to >16	37.1	32	>32	0.03 to >32	35.5	8	>8	≤0.12 to >8	35.5
YRIN (*n* = 44)	32	>32	≤0.015 to >32	36.4	8	16	0.06 to >16	29.5	>32	>32	0.03 to >32	27.3	8	>8	≤0.12 to >8	27.3
YRIK (*n* = 18)	1	>32	≤0.015 to >32	55.6	0.5	16	0.06 to >16	55.6	1	>32	0.06 to >32	55.6	2	>8	≤0.12 to >8	55.6
167 (*n* = 33)	8	>32	≤0.015 to >32	51.5	4	16	0.06 to >16	42.4	32	>32	0.03 to >32	42.4	4	>8	≤0.12 to >8	42.4
167 PBP3 (*n* = 23)	32	>32	≤0.015 to >32	30.4	8	16	0.12 to >16	17.4	32	>32	0.03 to >32	17.4	8	>8	≤0.12 to >8	17.4
361 (*n* = 10)	0.03	32	≤0.015 to >32	80	0.12	8	0.06 to 16	80	0.03	>32	0.03 to >32	80	≤0.12	8	≤0.12 to >8	80
361 PBP3 (*n* = 7)	0.03		≤0.015 to >32	71.4	0.12		0.06 to 16	71.4	0.06		0.03 to >32	71.4	≤0.12		≤0.12 to >8	71.4
405 (*n* = 18)	≤0.015	>32	≤0.015 to >32	83.3	0.12	16	0.06 to 16	83.3	0.06	>32	0.03 to >32	83.3	≤0.12	>8	≤0.12 to >8	83.3
405 PBP3 (*n* = 9)	1		≤0.015 to >32	66.7	0.5		0.06 to 16	66.7	1		0.06 to >32	66.7	2		≤0.12 to >8	66.7
410 (*n* = 20)	0.25	>32	≤0.015 to >32	55	0.5	16	0.06 to >16	55	1	>32	≤0.015 to >32	50	2	>8	≤0.12 to >8	50
410 PBP3 (*n* = 15)	16	>32	0.03 to >32	46.7	4	16	0.06 to >16	46.7	16	>32	0.06 to >32	40	4	>8	≤0.12 to >8	40
617 (*n* = 16)	≤0.015	>32	≤0.015 to >32	75	0.06	>16	0.06 to >16	75	0.03	>32	≤0.015 to >32	75	≤0.12	>8	≤0.12 to >8	75
617 PBP3 (*n* = 5)	>32		0.12 to >32	20	16		1 to >16	20	>32		0.25 to >32	20	>8		1 to >8	20
648 (*n* = 27)	0.03	>32	≤0.015 to >32	88.9	0.12	8	0.06 to >16	88.9	0.03	>32	0.03 to >32	85.2	0.25	>8	≤0.12 to >8	88.9
648 PBP3 (*n* = 3)	>32		>32 to >32	0	8		8 to >16	0	>32		>32 to >32	0	>8		>8 to >8	0

^*a*^ MIC_90_ values are not reported (not determined) for subgroups containing fewer than 10 isolates, as an MIC_90_ is not considered meaningful for *n* < 10.

^*b*^ %S, percentage susceptible applying EUCAST. Cefepime breakpoints were applied to cefepime-taniborbactam for comparison purposes.

Differences in the susceptibility of β-lactam and BL/BLI agents were also noted when comparing isolates harboring YRIN and YRIK insertions. YRIK-harboring isolates displayed lower susceptibility rates to aztreonam-avibactam (66.7% susceptible) when compared to overall isolates (90.3%), those carrying PBP3 insertions (80.6% susceptible), or isolates harboring YRIN insertions (86.4% susceptible). Conversely, YRIN-carrying isolates displayed lower susceptibility rates to cefiderocol (61.4% susceptible vs 69.4% for all PBP3-harboring isolates and 88.9% for YRIK isolates), cefepime-taniborbactam (4.5% vs 4.8% and 5.6%), ceftazidime-avibactam (36.4% vs 41.9% and 55.6%), meropenem-vaborbactam (36.4% vs 41.9% and 55.6%), imipenem-relebactam (29.5% vs 37.1% and 55.6%), and meropenem and imipenem (27.3% vs 35.5% and 55.6% for both).

The overall higher resistance rates observed for the isolates harboring YRIK insertions did not translate to all STs carrying these insertions. PBP3 YRIK insertions were noted only in STs 405, 410, and 617. The susceptibility rates of isolates belonging to ST405 and ST410 were consistently higher when compared to isolates belonging to ST617 (Table 2). Isolates with PBP3 insertions belonging to STs 405 and 410 displayed 100.0% and 80.0% susceptibility to aztreonam-avibactam, respectively, and susceptibility rates ranging from 40.0% to 100.0% to other agents. Meanwhile, isolates belonging to ST617 were all resistant to aztreonam-avibactam and had lower susceptibility rates to other agents, ranging from 20.0% to 40.0%. Among the five ST617 isolates harboring YRIN or YRIK alterations, four carried *bla*_NDM-5_ along with other β-lactamase genes, including *bla*_CMY-42_ in one isolate. This gene and *bla*_CMY-145_ that was found in the remaining isolate have been associated with aztreonam-avibactam resistance. Notably, three isolates belonging to ST617 possessing PBP3 insertions did not harbor CMY- or NDM-encoding genes and displayed aztreonam-avibactam MIC values at 0.5 mg/L. These isolates carried either *bla*_CTX-M-15_ or *bla*_CTX-M-55_ and displayed OmpC deletions at positions 182–183, 187, and 311–313. OmpF mutations (E224A, S225Q, S226P, and K229N) were also observed, but AcrR was wild type. The 11 ST617 isolates that possessed PBP3 WT sequences did not carry carbapenemases or CMY enzymes and were all susceptible to all BL/BLIs and carbapenems (data not shown).

A trend toward the lowest susceptibility rates was also observed for each ST included in this analysis when comparing overall isolates and the ones harboring PBP3 inserts (Table 2). *E. coli* isolates belonging to ST167, ST361, and ST648 displayed only PBP3 YRIN insertions, and aztreonam-avibactam inhibited 91.3%, 71.4%, and 100.0% of these isolates, respectively. Cefiderocol inhibited 56.5%, 100.0%, and 66.7% of the isolates belonging to ST167, ST361, and ST648, and cefepime-taniborbactam inhibited 8.7%, 0.0%, and 0.0%, respectively. All other agents displayed limited or no activity against isolates from ST167 (17.4%–30.4% susceptible) and ST648 (0.0% susceptible), but all inhibited 71.4% of the isolates belonging to ST361.

When stratified by the presence of β-lactamase genes and PBP3 insertions, the activity of aztreonam-avibactam, cefiderocol, cefepime-taniborbactam, and in some cases, cefepime-zidebactam was expectedly reduced for isolates harboring PBP3 insertions when compared to PBP3 WT isolates (Fig. 2). This observation was especially apparent for isolates carrying *bla*_CMY-42_, which has been associated with aztreonam-avibactam resistance. In the absence of the PBP3 insertion, isolates harboring *bla*_CMY-42_ had low MIC values for aztreonam-avibactam (0.25 mg/L) and other agents (0.06–0.25 mg/L) when compared to isolates carrying those insertions: 4 to >16 mg/L for aztreonam-avibactam and 0.25 to >32 mg/L for other agents. All isolates harboring *bla*_CMY-145_, previously associated with aztreonam-avibactam resistance, displayed PBP3 insertions. The five isolates carrying CMY-145 and a YRIN PBP3 insertion displayed aztreonam-avibactam MIC values ranging from 2 to 8 mg/L, and two out of five were susceptible to this BL/BLI. The single isolate harboring a YRIK PBP3 insertion displayed an aztreonam-avibactam MIC at 8 mg/L.

Overall, the presence of specific β-lactamase genes, combined with PBP3 YRIN alterations, did not consistently generate elevated MIC values. For example, isolates carrying *bla*_NDM-5_ with YRIN PBP3 insertions displayed aztreonam-avibactam MIC values ranging from very low (≤0.03 mg/L) to highly resistant (>16 mg/L). Cefiderocol and cefepime-taniborbactam displayed MIC values ranging from 2 to >64 mg/L and from 8 to >32 mg/L against these isolates, respectively. Similar results were observed for many other genes detected.

## DISCUSSION

Aztreonam-avibactam exhibits potent activity against Enterobacterales (12–14), CREs (16, 17), and MBL-producers (15); however, resistance to this BL/BLI combination has been documented. Aztreonam-avibactam resistance is widely regarded as a multifactorial phenomenon, but most resistant *E. coli* isolates reported harbor duplications in *fstI* that encode the YRIN or YRIK insertions in the PBP3 structure (6, 8, 9, 11, 23, 25). The prevalence and clinical relevance of these PBP3 insertion variants have been the subject of several investigations. While some studies report high rates in specific geographic regions (26, 27), a recent comprehensive analysis of *E. coli* genomes showed that YRIN and YRIK insertions occurred in only 1.9% of sequences from human, animal, and environmental sources. Isolates harboring these insertions were largely concentrated within a few high-risk clones, including ST410, ST167, and ST405 (18). These lineages are considered successful bacterial clones that play pivotal roles in the global spread of antimicrobial resistance genes (28), including ESBLs (29), carbapenemases (30), and CMY-type enzymes (28). As demonstrated in evolutionary studies of ST410 (28), the isolates from these high-risk lineages acquired these resistance genes over time and accumulated genomic mutations, including the PBP3 insertions and alterations in outer membrane proteins.

We initiated this study to examine the occurrence and phenotypic impact of PBP3 insertions among *E. coli* isolates belonging to six high-risk sequence types (STs 167, 361, 405, 410, 617, and 648). These lineages were detected in approximately 8% of a large collection of isolates displaying resistance to broadly used β-lactam agents. Among 124 isolates belonging to these high-risk clones, 62 harbored the PBP3 insertions YRIN and YRIK. Overall, isolates with altered PBP3-encoding genes were more resistant to β-lactams, but differences in the susceptibility profiles of isolates harboring YRIN versus YRIK insertions were noted. Isolates possessing YRIK insertions were less common but more resistant to aztreonam-avibactam. By contrast, isolates harboring YRIN were more common and exhibited lower susceptibility rates to several other β-lactams and BL/BLI combinations but were more susceptible to aztreonam-avibactam when compared to isolates with YRIK insertions. Furthermore, isolates belonging to ST617 containing PBP3 insertions were more resistant to aztreonam-avibactam and cefiderocol when compared to other STs, regardless of the PBP3 insertion type and other resistance mechanisms, suggesting lineage-specific contributions to resistance phenotypes.

The presence of certain β-lactamases has also been implicated in aztreonam-avibactam resistance. CMY enzymes—particularly CMY-42, CMY-141, and CMY-145—combined with PBP3 insertions have been identified among aztreonam-avibactam resistant isolates (9, 10, 23, 24). CMY-42 seems not as well inhibited by avibactam when compared to CMY-2. When genes encoding these enzymes were expressed in the same genetic background, the strains carrying *bla*_CMY-42_ displayed twofold higher MIC values for aztreonam when compared to *bla*_CMY-2_, but the aztreonam-avibactam MIC results were fourfold higher (9). In our collection, 13 isolates harbored CMY-42 (five isolates), CMY-141 (two isolates), or CMY-145 (six isolates). Remarkably, four of these isolates were susceptible to aztreonam-avibactam, including one carrying *bla*_CMY-42_ with a WT PBP3 sequence, suggesting that the production of CMY-42 alone is not singularly responsible for aztreonam-avibactam resistance.

Our findings reinforce that although PBP3 insertions are common within certain globally disseminated *E. coli* lineages associated with β-lactam resistance, their presence alone is not a reliable determinant of reduced susceptibility to aztreonam-avibactam. The observation that many isolates harboring YRIK or YRIN insertions—including some co-producing CMY variants or NDM enzymes—remained susceptible to this BL/BLI combination underscores the multifactorial nature of resistance. PBP3 insertions require additional factors such as β-lactamase activity with low avibactam affinity, permeability impairment, efflux overexpression, and potentially as yet undiscovered lineage-specific factors in order to confer resistance to aztreonam-avibactam.

Additionally, a recent study supports that PBP3 insertions do not easily emerge upon aztreonam-avibactam exposure. Single-step mutants were generated by exposing multiple Enterobacterales species with different β-lactamase backgrounds to aztreonam-avibactam (31). Most of the mutants generated display alterations in membrane stress systems such as BaeSR and EnvZ and rarely harbored PBP3 alterations. This observation, together with the rates of resistance emergence, led the authors to conclude that the risk of aztreonam-avibactam resistance emerging *de novo* was lower than for ceftazidime-avibactam. Intriguingly, all isolates with YRIN PBP3 insertions characterized in this study also possessed E349K and I532L substitutions, alterations not observed in isolates lacking this insertion. Similarly, all isolates with YRIK insertions also possessed an A413V substitution, which was observed only in two isolates without this insertion. These commonly associated alteration profiles may suggest that when borne alone, YRIK/YRIN insertions result in some reduction of fitness, for which subsequent compensatory mutations are required. Alternatively, the presence of the A413V in the absence of YRIK could also suggest these non-insertion mutations serve as stepping stones to more susceptibility-affecting insertions. Further studies using isogenic strains could be performed to investigate these findings. Nonetheless, the dissemination of high-risk *E. coli* clones enriched for these insertions remains an epidemiological concern that requires continued monitoring and further understanding.

Despite the high prevalence of PBP3 insertions within several high-risk lineages, aztreonam-avibactam retained activity against more than 90% of these clonal isolates and over 80% of isolates carrying PBP3 insertions. This BL/BLI exhibited higher activity than all clinically available comparator agents. Overall, this study supports the observation that PBP3 insertions contribute to β-lactam resistance but should not be viewed as a surrogate marker of aztreonam-avibactam resistance. Instead, phenotypic susceptibility testing remains essential to accurately assess the activity of aztreonam-avibactam. This is further highlighted by the recent limitations reporting that the aztreonam plus ceftazidime-avibactam broth-disk elution test failed to detect the majority of *E. coli* isolates carrying PBP3 insertions (32).

## SUPPLEMENTAL MATERIAL

Supplemental tables aac.00129-26-s0001.docx Tables S1 and S2.

ASM does not own the copyrights to Supplemental Material that may be linked to, or accessed through, an article. The authors have granted ASM a non-exclusive, world-wide license to publish the Supplemental Material files. Please contact the corresponding author directly for reuse.
