# Supplementary material for: Impact of PBP3 insertions and β-lactamase-encoding genes on the susceptibility of Escherichia coli isolates belonging to high-risk clones against aztreonam-avibactam and comparator agents
Source: Antimicrob Agents Chemother. 2026 Sep 9;70(10):e00129-26. doi: 10.1128/aac.00129-26 (PMC13688841; doi:10.1128/aac.00129-26)
Supplement: Supplementary file 1 [file aac.00129-26-s0001.docx]

**Supplemental Table 1** PBP3 insertions YRIN and YRIK and other PBP3 alterations among *E. coli* isolates belonging to STs 167, 361, 405, 410, 617, and ST648

|  |  | **No. of isolates overall and by ST** | | | | | | |
| --- | --- | --- | --- | --- | --- | --- | --- | --- |
| **PBP3 insertions** | **PBP3 alterations** | **All isolates** | **ST167** | **ST361** | **ST405** | **ST410** | **ST617** | **ST648** |
| YRIK | E149D, T233A, V332I, A413V | 18 |  |  | 9 | 5 | 4 |  |
| YRIN | E149D, Q227H, T233A, V332I, E349K, I532L | 43 | 23 | 7 |  | 9 | 1 | 3 |
| YRIN | T75A, E149D, Q227H,T233A,V332I, E349K, I532L | 1 |  |  |  | 1 |  |  |
| WT | E149D, Q227H, T233A, V332I | 12 | 1 |  |  |  | 11 |  |
| WT | E149D, T233A | 8 |  |  | 8 |  |  |  |
| WT | E149D, T233A, A413V | 1 |  |  | 1 |  |  |  |
| WT | E149D, T233A, V332I | 16 | 9 | 3 |  | 4 |  |  |
| WT | E149D, T233A, V332I, A413V | 1 |  |  |  | 1 |  |  |
| WT | T233A | 20 |  |  |  |  |  | 20 |
| WT | T233A, A317T | 1 |  |  |  |  |  | 1 |
| WT | T233A, A317V | 1 |  |  |  |  |  | 1 |
| WT | T233A, L369F | 1 |  |  |  |  |  | 1 |
| WT | T233A, S473I | 1 |  |  |  |  |  | 1 |

**Supplemental Table 2** Acquired β-lactamases enzyme arrays detected among *E. coli* isolates belonging to STs 167, 361, 405, 410, 617 and 648 with stratification by PBP3 insertions YRIN and YRIK. Amino acid substitutions relative to the respective reference enzyme are indicated in parentheses after the enzyme name; positions are given as the wild-type residue, the codon number, and the variant residue (for example, M102I denotes a methionine-to-isoleucine change at position 102), and X denotes a nonsense (premature stop) codon resulting in a truncated protein (for example, Q55X denotes a glutamine-to-stop change at position 55). WT, wild-type PBP3 sequence at the insertion site; YRIN and YRIK indicate the four-amino-acid PBP3 insertions

| **Acquired β-lactamases** | **No. of isolates overall, by sequence type (ST) and PBP3 insertion** | | | | | | | | | | | | | | | | | | | |
| --- | --- | --- | --- | --- | --- | --- | --- | --- | --- | --- | --- | --- | --- | --- | --- | --- | --- | --- | --- | --- |
|  | **All** | **ST167** | |  | **ST361** | |  | **ST405** | |  | **ST410** | | |  | **ST617** | | |  | **ST648** | |
|  |  | **YRIN** | **WT** |  | **YRIN** | **WT** |  | **YRIK** | **WT** |  | **YRIK** | **YRIN** | **WT** |  | **YRIK** | **YRIN** | **WT** |  | **YRIN** | **WT** |
| CMY-141, CTX-M-15, OXA-1 | 1 |  |  |  |  |  |  |  |  |  | 1 |  |  |  |  |  |  |  |  |  |
| CMY-141, CTX-M-3, OXA-1 | 1 |  |  |  |  |  |  |  |  |  | 1 |  |  |  |  |  |  |  |  |  |
| CMY-145 (Q55X), CTX-M-15 | 1 |  |  |  |  |  |  |  |  |  |  |  |  |  | 1 |  |  |  |  |  |
| CMY-145, NDM-5, OXA-1(M102I), TEM-1 | 1 |  |  |  | 1 |  |  |  |  |  |  |  |  |  |  |  |  |  |  |  |
| CMY-145, NDM-5, TEM-1 | 2 | 2 |  |  |  |  |  |  |  |  |  |  |  |  |  |  |  |  |  |  |
| CMY-145, NDM-7 | 1 | 1 |  |  |  |  |  |  |  |  |  |  |  |  |  |  |  |  |  |  |
| CMY-145, OXA-244 | 1 | 1 |  |  |  |  |  |  |  |  |  |  |  |  |  |  |  |  |  |  |
| CMY-2 | 1 |  |  |  |  |  |  |  |  |  |  |  |  |  |  |  |  |  |  | 1 |
| CMY-2, CMY-42, OXA-181, OXA-1, TEM-1 | 1 |  |  |  |  |  |  |  |  |  |  | 1 |  |  |  |  |  |  |  |  |
| CMY-2, CTX-M-14, CTX-M-15, NDM-5, OXA-1 | 1 |  |  |  |  |  |  |  |  |  |  |  |  |  | 1 |  |  |  |  |  |
| CMY-2, CTX-M-15, NDM-5, OXA-1, TEM-1 | 4 |  |  |  |  |  |  |  |  |  |  | 4 |  |  |  |  |  |  |  |  |
| CMY-2, CTX-M-15, NDM-5, OXA-181, OXA-1, TEM-1 | 2 |  |  |  |  |  |  |  |  |  |  | 2 |  |  |  |  |  |  |  |  |
| CMY-2, CTX-M-15, OXA-1, TEM-1 | 1 |  |  |  |  |  |  |  |  |  |  | 1 |  |  |  |  |  |  |  |  |
| CMY-2, TEM-1 | 2 |  |  |  |  |  |  |  | 1 |  |  |  |  |  |  |  |  |  |  | 1 |
| CMY-4, CTX-M-55 | 1 |  |  |  |  |  |  |  | 1 |  |  |  |  |  |  |  |  |  |  |  |
| CMY-42 | 1 |  |  |  |  |  |  |  |  |  |  |  |  |  |  |  |  |  |  | 1 |
| CMY-42, CTX-M-15, OXA-181, OXA-1, TEM-1 | 1 |  |  |  |  |  |  |  |  |  |  | 1 |  |  |  |  |  |  |  |  |
| CMY-42, NDM-5 | 1 |  |  |  |  |  |  |  |  |  |  |  |  |  |  | 1 |  |  |  |  |
| CMY-42, TEM-1 | 1 |  |  |  | 1 |  |  |  |  |  |  |  |  |  |  |  |  |  |  |  |
| CMY-6, NDM-1, OXA-1 | 1 |  |  |  |  |  |  |  |  |  |  |  | 1 |  |  |  |  |  |  |  |
| CTX-M-14 | 6 |  |  |  |  |  |  |  |  |  |  |  |  |  |  |  | 2 |  |  | 4 |
| CTX-M-14, KPC-2 | 1 |  |  |  |  |  |  |  |  |  |  |  |  |  |  |  |  |  |  | 1 |
| CTX-M-14, OXA-48, TEM-1 | 1 |  | 1 |  |  |  |  |  |  |  |  |  |  |  |  |  |  |  |  |  |
| CTX-M-14, TEM-1 | 1 |  |  |  |  |  |  |  |  |  |  |  |  |  |  |  |  |  |  | 1 |
| CTX-M-15 | 13 | 2 | 1 |  |  |  |  | 3 |  |  |  |  |  |  |  |  | 3 |  |  | 4 |
| CTX-M-15, DHA-1 | 1 |  |  |  |  |  |  |  |  |  |  |  |  |  |  |  |  |  |  | 1 |
| CTX-M-15, DHA-1, OXA-181 | 1 |  |  |  |  |  |  | 1 |  |  |  |  |  |  |  |  |  |  |  |  |
| CTX-M-15, NDM-5 | 2 |  |  |  |  |  |  | 2 |  |  |  |  |  |  |  |  |  |  |  |  |
| CTX-M-15, NDM-5, OXA-1 | 15 | 10 |  |  | 1 |  |  |  |  |  | 2 |  |  |  | 2 |  |  |  |  |  |
| CTX-M-15, NDM-5, OXA-1, TEM-1 | 7 | 3 |  |  |  |  |  | 1 |  |  |  |  |  |  |  |  |  |  | 3 |  |
| CTX-M-15, NDM-5, OXA-181, OXA-1 | 2 | 2 |  |  |  |  |  |  |  |  |  |  |  |  |  |  |  |  |  |  |
| CTX-M-15, OXA-1 | 10 |  |  |  |  |  |  |  | 3 |  | 1 |  |  |  |  |  | 3 |  |  | 3 |
| CTX-M-15, OXA-1(M102I), TEM-1 | 1 |  |  |  | 1 |  |  |  |  |  |  |  |  |  |  |  |  |  |  |  |
| CTX-M-15, OXA-1, OXA-48 | 1 |  |  |  |  |  |  | 1 |  |  |  |  |  |  |  |  |  |  |  |  |
| CTX-M-15, OXA-1, TEM-1 | 6 |  |  |  |  |  |  |  |  |  |  |  |  |  |  |  | 2 |  |  | 4 |
| CTX-M-15, OXA-1, TEM-1, TEM-57 | 1 |  |  |  |  | 1 |  |  |  |  |  |  |  |  |  |  |  |  |  |  |
| CTX-M-15, OXA-181, OXA-1 | 1 |  |  |  |  |  |  |  |  |  |  |  |  |  |  |  |  |  |  | 1 |
| CTX-M-15, OXA-48 | 1 |  |  |  |  |  |  | 1 |  |  |  |  |  |  |  |  |  |  |  |  |
| CTX-M-15, TEM-1 | 11 |  | 3 |  |  |  |  |  | 4 |  |  |  | 3 |  |  |  |  |  |  | 1 |
| CTX-M-15, TEM-1(V78I) | 1 |  |  |  |  |  |  |  |  |  |  |  |  |  |  |  |  |  |  | 1 |
| CTX-M-27, TEM-40 | 1 |  | 1 |  |  |  |  |  |  |  |  |  |  |  |  |  |  |  |  |  |
| CTX-M-55 | 5 | 1 | 2 |  |  | 1 |  |  |  |  |  |  |  |  |  |  | 1 |  |  |  |
| CTX-M-55, OXA-1(M102I), TEM-1(M1L) | 2 |  |  |  | 2 |  |  |  |  |  |  |  |  |  |  |  |  |  |  |  |
| CTX-M-55, TEM-1 | 2 |  | 1 |  |  | 1 |  |  |  |  |  |  |  |  |  |  |  |  |  |  |
| CTX-M-55, TEM-1(M1L) | 1 |  |  |  | 1 |  |  |  |  |  |  |  |  |  |  |  |  |  |  |  |
| DHA-1, NDM-19, TEM-1 | 1 | 1 |  |  |  |  |  |  |  |  |  |  |  |  |  |  |  |  |  |  |
| DHA-1, TEM-1 | 1 |  | 1 |  |  |  |  |  |  |  |  |  |  |  |  |  |  |  |  |  |
| KPC-2, OXA-1, TEM-1 | 1 |  |  |  |  |  |  |  |  |  |  | 1 |  |  |  |  |  |  |  |  |
| TEM-176 | 1 |  |  |  |  |  |  |  |  |  |  |  | 1 |  |  |  |  |  |  |  |
